# Learning Choreography: An Investigation of Motor Imagery, Attentional Effort, and Expertise in Modern Dance

**DOI:** 10.3389/fpsyg.2019.00422

**Published:** 2019-03-01

**Authors:** Katy Carey, Aidan Moran, Brendan Rooney

**Affiliations:** School of Psychology, University College Dublin, Dublin, Ireland

**Keywords:** dance, creativity, expertise, motor imagery, attention, pupillometry

## Abstract

The study of choreography in dance offers researchers an intriguing window on the relationship between expertise, imagination, and attention in the creative process of learning new movements. The present study investigated an unresolved issue in this field – namely, the effects of expertise on motor imagery (MI; or the mental rehearsal of actions without engaging in the actual movements involved) and attentional effort (as measured by pupil dilation) on dancers while they engaged in the processes of learning, performing, and imagining a dance movement. Participants were 18 female dancers (mean age = 23, *SD* = 5.85) comprising three experience levels (i.e., novice, intermediate and expert performers) in this field. Data comprised these participants’ MI scores as well as their pupil dilation while they learned, performed, and imagined a 15 s piece of choreography. In addition, the time taken both to perform and to imagine the choreography were recorded. Results showed no significant effect of dance expertise on MI but some differences between beginners and intermediate dancers in attentional effort (pupil dilation) at the start of the performance and the imagined movement conditions. Specifically, the beginners had the highest pupil dilation, with the experts having the second highest, while intermediates had the lowest dilation. Further analysis suggested that the novice dancers’ pupil dilation at the start of the performance may have been caused, in part, by the initial mental effort required to assess the cognitive demands of the dance task.

## Introduction

Dance is a form of artistic expression and communication involving “moving the body through time and space” (Cross and Ticini, 2012, p. 6). It is a cognitively and physically demanding art-form which elicits creativity in the dancer, who is required to be able to adapt movements that are rhythmical and esthetically pleasing (Kaufman and Baer, 2005). Since the early 2000s, it has attracted research attention from psychologists and neuroscientists because it provides a “real life” window into topics like expertise (the study of what makes people exceptionally knowledgeable about, or skilled in, a particular domain; Moran and Toner, 2017), embodied cognition (the theory that cognition is largely grounded in sensorimotor experience; Laakso, 2011) and creativity (the capacity to produce ideas and outputs that are novel and adaptive or functional; Simonton and Damian, 2013). Dance research has addressed both theoretical and practical issues. For example, at a theoretical level, Cross et al. (2014) showed how the neuroscientific study of dance can elucidate the mechanisms by which the brain perceives and learns complex motor sequences. In addition, research on dance facilitates the study of inter-genre differences in creativity among performers. For example, Fink and Woschnjak (2011) discovered that experienced modern contemporary dancers have heightened figural and verbal creative abilities in comparison to dancers of other genres (such as ballet and jazz) as well as non-dancers. At an applied level, research on dancers’ mental rehearsal techniques has provided fascinating insights into the cognitive process of “motor imagery” (MI) or “mentally simulating an intended action without actually producing it” (Smith and Kosslyn, 2007, p. 456). For example, Nordin and Cumming (2005) conducted in-depth interviews with professional dancers to find out where, when and why they used MI. One of their findings was that dancers reported using imagery in practice both as a “creative tool” (p. 401) and to help them to learn and remember steps. Furthermore, Kaufman and Baer (2005) argued that dancers are inherently creative due to the constant decision making that they require when improvising or creating their own choreography and also when learning and performing movements. When learning a new movement (i.e., one that is not in their behavioral repertoire), dancers are confronted with a problem. In such situations, according to Weisberg’s (2018) “expertise view” of creativity, the “presentation of a problem results in retrieval of knowledge – i.e. expertise – from memory; *creative advances evolve out of attempts to apply that knowledge to the new situation*” (p. 813; italics ours). Interestingly, Kaufman and Baer (2005) also postulated that a creative dancer is one who can utilize MI to achieve a heightened awareness of performance, as images can incorporate physicality, emotion and expressiveness, which are three key components of a dance performance.

Unfortunately, despite the preceding research, little or nothing is known at present about the relationship between dancers’ expertise and their use of cognitive processes such as MI and attention (or “focusing on specific features, objects or locations or on certain thoughts or activities”; Goldstein, 2011, p. 391) when attempting to master new choreography. According to Torrents et al. (2015), learning choreography elicits “possibility thinking” in dancers – a creative process that begins with artistic performers imaginatively asking “what if?” before they proceed to execute a novel action. Similarly, Kaufman and Baer (2005) state that throughout a performance, the dancer faces moment-by-moment decisions such as how and when to execute movements, making it an inherently creative process. Against this background, and in view of the dearth of research on cognitive psychological aspects of dance, the purpose of the present paper is to investigate the relationship between MI and attentional effort (or the allocation of mental resources to satisfy cognitive demands; Sarter et al., 2006) in dancers of differing expertise who engage in this possibility thinking while learning and performing choreography. Before explaining MI and attentional effort in more detail, however, it is important to understand the methodological approach that we have adopted in the present paper: namely, “process tracing” – a term borrowed from Williams and Ericsson (2005) to refer to procedures (e.g., eye tracking technology or the computerized measurement of the location, duration and sequence of people’s visual fixations when they inspect a given scene) that help to identify the processes or mechanisms underlying expert performance in a given field. As Ericsson (2018) put it, process tracing “is essential for uncovering detailed information about most of the important characteristics that are responsible for the superiority of … experts’ achievements” (p. 207). To the best of our knowledge, no previous published study has used this “process tracing” approach to investigate the learning, performing and mental simulation of choreographed dance movements and the potential creative thinking which underlies these processes.

### Exploring Creativity in Dance – Toward a Process Tracing Approach

In order to explain the “process tracing” approach to the study of dance, we need to consider how creativity is approached in psychology. According to Simonton and Damian (2013), creativity can be studied from at least three different perspectives in psychology: namely, those of products, persons, or processes. Research on creative *products* focuses mainly on the “ideational development” that spawns creative outputs such as poems or paintings. Next, research on creative *persons* typically explores either how the originators concerned manage to acquire relevant domain-specific expertise or how their cognitive abilities (e.g., divergent thinking skills) and inclinations (e.g., cognitive style) facilitate the outputs under scrutiny. Finally, research on creative *processes* is largely concerned with identifying and tracing the neurocognitive mechanisms that are postulated to mediate creative thought or action. In this latter regard, a variety of methodological tools is available for this type of “process tracing” of psychological mechanisms. For example, electroencephalography (EEG; a technique that measures cortical activity by recording electrical signals generated by the brain using non-invasive electrodes placed at different points on the scalp in an elastic cap) has been used to explore the neural signature of creativity in dance. Thus, Fink et al. (2009) compared alpha-wave activity in expert professional dancers with that of a group of relative novices. Some notable differences were evident. For example, during a creative improvisation dance task, the professional performers displayed more right-hemispheric alpha synchronization in posterior parietal regions than did the novices. Alpha frequency EEG activity appears to be especially sensitive to creativity-related cognitive demands. Thus, synchronization of alpha has been observed to be stronger in response to tasks of creative thinking (such as generating unusual uses of everyday objects) in comparison with tasks requiring more convergent thinking (Fink et al., 2007). Interestingly, synchronization of alpha wave activity has been shown to increase as a result of creative thinking training (Fink et al., 2006). But do conventional teaching methods actually encourage a dancer’s creative skills? Doubts about this issue were raised by Chappell et al. (2009) who found that because of increasing pressure on dancers to reach prescribed levels of attainment, certain formulaic styles of teaching, and choreography have become popular in dance education. Unfortunately, these teaching styles may hinder a dancer’s ability to generate movement solutions to the motor problems confronting them. To circumvent such difficulties, researchers have investigated the efficacy of novel teaching approaches on creativity in dance. For example, Torrents et al. (2015) discovered that when specific constraints were deliberately placed on dancers while improvising (e.g., by requiring them to keeping one body-part in a designated position), the originality of their subsequent movement (as assessed by expert performers/choreographers) actually improved. Augmenting these studies, evidence has emerged to show that dance learners’ mental imagery processes can facilitate the creative process of acquiring, or learning, new movements (Nordin and Cumming, 2007; Overby and Dunn, 2011; Heiland and Rovetti, 2013). This discovery leads us to consider the key variables in the present study – namely, MI and attentional effort.

### Motor Imagery: Nature, Measurement, and Mechanisms

As mentioned earlier, motor imagery (MI: also known as “motor imagination”; Hanakawa, 2016) is the cognitive simulation of an action without actually executing it (see review by Moran et al., 2012). Research interest in MI is as old as the discipline of psychology itself. To illustrate, James (1890), in his prescient discussion of “motor images” (p. 708), suggested somewhat counter-intuitively that by anticipating experiences imaginatively, people actually learn to skate in the *summer* and to swim in the *winter.* Since the 1890s, hundreds of experimental studies have demonstrated the efficacy of MIP in improving skill-learning in a variety of performance domains (Moran et al., 2012). MI can be assessed using either subjective or objective measures. Whereas the former measures include psychometric instruments that require respondents to rate some aspect of their imagery experience (e.g., its vividness or clarity), the latter assess proficiency in imagery skills through the accuracy or speed with which respondents solve problems or complete tasks known to require imagery ability. A recent subjective measure of MI is the Movement Imagery Questionnaire-3 (MIQ-3; Williams et al., 2012) – which is an updated version of the movement imagery questionnaire (MIQ; Hall and Pongrac, 1983). The MIQ-3 is a 12-item questionnaire that assesses the ease or difficulty of generating images of four different movements (i.e., knee lift, jump, arm movement, and waist bend) from different imagery perspectives. For each item, participants are required to read a description of the movement, physically perform the movement, and then imagine that movement from the designated perspective. Respondents are then required to rate the resultant image on a 7-point Likert scale ranging from 1 (*very hard to see/feel*) to 7 (*very easy to see/feel*). Subscale scores range from 4 to 28 and higher scores reflect stronger imagery ability. According to its developers, the MIQ-3 displays good internal consistency. Turning to objective measures of MI, two main options are available at present. On the one hand, Madan and Singhal (2013, 2014) developed the test of ability in movement imagery (TAMI) which requires respondents to imagine a series of bodily movements and then to select the correct option from a set of possible body-positioning images – including the appropriate one. Alternatively, MI can be measured objectively by comparing the time required to execute and imagine specific actions. To explain the rationale for this approach, if imagined and executed actions rely on similar motor representations and activate some common brain areas (as predicted by the “functional equivalence” hypothesis; discussed below), then their *temporal* organization should be equivalent. Accordingly, there should be a close correspondence between the time required to *mentally* perform a given action and that required for its *actual* execution. So, “mental chronometry” tasks measure MI by evaluating the correspondence between the actual and imagined duration required to perform a given action (see review by Guillot and Collet, 2005). Collet et al. (2011) also discussed factors which may mediate the correspondence between real and imagined movements, such as level of experience with the movement in question or the type of image the individual has generated, e.g., visual versus kinaesthetic.

Although there is a dearth of studies evaluating MIP programs in dancers (see Abraham et al., 2017), a growing research literature exists on other aspects of dance imagery (see reviews by Overby and Dunn, 2011; Pavlik and Nordin-Bates, 2016; Fisher, 2017). For example, Pavlik and Nordin-Bates (2016) reviewed 43 papers on dance imagery that had been published between 1990 and 2014. They concluded that dancers tend to use “technique imagery” (or mental rehearsal of movements or sequences) more frequently than other types of imagery – especially “to picture spatial relationships while simultaneously stimulating creativity and helping to plan the next steps” (p. 56). Interestingly, choreographers often use imagery to solve problems within a dance piece (Nordin and Cumming, 2005). In addition, Pavlik and Nordin-Bates (2016) concluded that dancers tend to use imagery before, during and after class, rehearsal, and performance. Other studies have examined the imagery abilities of dancers. Thus, Overby (1990) reported that experienced dancers tend to have stronger imagery abilities than novices – but, curiously, not for MI (as assessed by the Movement Imagery Questionnaire, MIQ; Hall and Pongrac, 1983). A possible explanation for this anomaly is that the MIQ is not an objective measure of MI. Subsequently, Jola and Mast (2005) compared the imagery abilities of dancers with those of non-dancers. Results showed that whereas dancers performed better than non-dancers on tests of imagined bodily rotation, they performed *worse* than non-dancers on tests assessing the rotation of inanimate objects - suggesting that dancers’ imagery superiority may be domain-specific. Interestingly, Jola and Mast’s (2005) study appears to be the only one in the dance imagery literature which used an objective measure of imagery (specifically, Shepard and Metzler’s 1988, mental rotation test). Clearly, therefore, there is an urgent need for dance research on MI to combine subjective and objective measures – as we have done in the present paper.

Before we conclude this section, however, it is important to consider the possible theoretical mechanisms by which MI works. Perhaps the most influential account of these mechanisms is that offered by motor simulation theory (MST; Jeannerod, 1994, 2001, 2006). According to MST (see critique by O’Shea and Moran, 2017), action planning and MI share a common mental representation. In other words, MI is based on the motor representation that underlies actual motor performance. Next, MST proposes that the motor system is part of a cognitive network that includes other psychological activities such as imagining actions, learning by observation, and attempting to understand the behavior of other people. Thirdly, Jeannerod (2001) claimed that actions involve a *covert* stage during which they are prepared or simulated mentally. This covert stage involves “a representation of the future, which includes the goal of the action, the means to reach it, and its consequences on the organism and the external world. Covert and overt stages thus represent a continuum, such that every overtly executed action implies the existence of a covert stage” (p. S103). Finally, combining these propositions, Jeannerod (2001) postulated that “MI … should involve, in the subject’s motor brain, neural mechanisms similar to those operating during the real action” (pp. S103-S104) – the so-called “functional equivalence” hypothesis. According to this hypothesis, imagined and executed actions share, to some degree, certain mental representations and underlying mechanisms (see brief review in Moran et al., 2012). For example, both overt and imagined actions share a motor representation of an intention to act. Whereas this intention is converted into an actual physical movement in the case of overt actions, it is inhibited in the case of imagined actions. Nevertheless, this shared motor representation facilitates certain forms of functional equivalence between actual and imagined actions. Thus, Hétu et al. (2013) found that the neural network underlying MI includes several cortical regions known to control actual motor execution, such as the premotor cortex, parietal cortex and fronto-parietal regions such as the basal ganglia, putamen and pallidum. Having examined the nature and measurement of MI, and some of its key neurocognitive mechanisms, let us now turn to the second important variable in the present study – attentional effort.

### Attentional Processes in Dance: Attentional Effort

The construct of attention has been invoked by cognitive psychologists for over a century to account for a range of mental phenomena such as selectivity of information processing, intensity of focus, and the allocation of limited mental resources to regulate concurrent task performance. Within attentional research, it has long been known that expert performance in any skilled domain depends significantly on the ability to focus selectively on task-relevant information (Moran, 1996). But apart from selectivity of information processing, another attentional process that seems crucial to skill learning is “attentional effort” (also known as “mental effort” or “cognitive effort”; Piquado et al., 2010; Burge et al., 2013). This rather loosely defined, if intuitively appealing, construct denotes the allocation of mental resources in order to satisfy task demands. For example, trying to multiply 36 by 49 in one’s head requires more cognitive exertion than does multiplying 6 by 9. So, attentional effort captures the *intensive*, as distinct from the selective, nature of cognitive resource allocation. To explain this distinction, Kahneman (1973) differentiated between “selective” and “intensive” aspects of attention. Whereas “selective” attention refers to the fact that we can assimilate only a fraction of all information available to us, “intensive” attention refers to the *intensity* with which one’s attention is focused in a particular situation. For Kahneman (1973), therefore, “the intensive aspect of attention corresponds to effort” (p. 12).

One way of assessing attentional effort is through “pupillometry” – or the measurement of task-evoked changes in the diameter of the pupil of the eye as a function of cognitive processing (Mathôt and Van der Stigchel, 2015). To explain, pupil size changes in response to three different kinds of stimuli (Mathôt, 2018). Specifically, it constricts in response to brightness, constricts in response to near fixation, and dilates in response to increased cognitive activity, such as increased levels of arousal or mental effort. For example, Hess and Polt (1964) showed that pupil size is a reliable indicator of mental effort and arousal. They asked participants to perform mental calculations of varying complexity (e.g., 7 × 8 was deemed easy, whereas 16 × 23 was regarded as difficult) and discovered that pupil size reflected the difficulty of the calculation. The harder the calculation was to perform, the larger the pupil. Although space limitations preclude a review of research on pupillometry (but see Mathôt and Van der Stigchel, 2015), pupil dilation effects have been demonstrated reliably for cognitive tasks involving multiplication problems (Hess and Polt, 1964), visual search (Porter et al., 2007), and change detection (Unsworth and Robison, 2015) tasks. Furthermore, mounting evidence suggests that the pupil remains dilated throughout the expenditure of cognitive load (Granholm et al., 1996). Unfortunately, apart from studies by Moran et al. (2016) and O’Shea and Moran (2016), pupillometry has rarely been investigated in sport, exercise and performance psychology despite its potential importance as a non-invasive, online measure of attentional effort. Clearly, as, Beatty and Lucero-Wagoner (2000) claimed, whatever activates the mind causes the pupil to dilate. According to Kahneman (1973), pupil dilation is “the best single index” (p. 18) of attentional effort. Supporting this view, recent evidence (e.g., Murphy et al., 2014) shows that pupil size predicts brain activity in the locus coeruleus-norepinephrine (LC-NE) system – the one that regulates the allocation of attentional resources to task engagement.

Some previous researchers have investigated attentional factors in dance. For example, Guss-West and Wulf (2016) surveyed a sample of expert ballet dancers to determine their preferred attentional focus while performing certain dance movements (e.g., *a pirouette en dehors*). Results showed that the dancers reported adopting either internal foci or a combination of internal and external foci most of the time when performing. Unfortunately, as this study relied on self-report data rather than objective measures, its results are limited to *perceived* rather than actual attentional processes. A different approach was adopted by Stevens et al. (2010) who used eye-tracking equipment to explore expert-novice differences in dancers’ visual fixations and eye movements when watching a contemporary dance film. The hypothesis under investigation was that dance experts’ expectations about dance would facilitate their perception of dance movements. Corroborating this hypothesis, Stevens et al. (2010) discovered that the fixation times of dance experts watching a dance film were significantly shorter than those of novice counterparts – presumably reflecting a cognitive advantage (superior pattern recognition skills and more accurate expectations) of the former over the latter performers. But what of the level of attentional effort required by the creative, possibility thinking involved in learning and performing choreography? While it is understood that attention and focus are imperative in facilitating creativity (e.g., Kasof, 1997), less is known specifically about attentional effort in a dance setting. Kaufman and Baer (2005) identified attentiveness as an essential factor in facilitating creativity as well as stating that “creative performers of movement are those who maintain heightened awareness of and sensitivity to the creativity of the human body” (p. 89). Although this claim appears to support the role of attention in these creative processes, there is a lack of research which specifically examines the relationship between attentional *effort* and learning and performing choreography.

### Unresolved Issues in Cognitive Psychological Research on Dance

From the preceding sections, it is evident that there are at least two major gaps in cognitive psychological research in dance. Firstly, few studies have examined the MI processes of dancers. Accordingly, the extent to which these processes vary with dancers’ level of expertise is unknown. Secondly, no published studies could be located in which the attentional effort of dancers was objectively investigated while they engage in the creative or “possibility thinking” (Torrents et al., 2015) process that is hypothesized to aid the learning, performing and imagining of a new piece of choreography. Therefore, the purpose of the present study was to address these objectives.

### The Present Study

The present study investigates the relationship between dancers’ MI ability, attentional effort, and dance expertise (at three levels: novice, intermediate, and expert performer) while they learned, performed and imagined a piece of dance choreography. In order to measure dancers’ MI abilities, we shall use a novel combination of subjective and objective measures described earlier – namely, the MIQ-3, the TAMI and the mental chronometry approach. Attentional effort will be assessed by the measurement of pupil dilation (as recorded by the Tobii Pro Glasses – a wearable eye-tracker; Tobii Technology, 2017).

## Hypotheses

Hypothesis 1: That dancers’ MI abilities will vary with their level of expertise.
Hypothesis 2: That the difference between actual and imagined time required to perform the choreography will vary indirectly with level of dance expertise – such that expert dancers will display the greatest congruence between actual and imagined time and that novices will display the lowest congruence between these times.
Hypothesis 3: That there will be a significant interaction between level of dance expertise and level of pupil dilation at three time-points throughout the learning, performing and imagined movement conditions.

## Materials and Methods

### Participants

Eighteen female ballet and modern dancers (*M* = 23 years; *SD* = 5.85) took part in this study, with 6 dancers recruited at each of three different levels of expertise (i.e., novice, intermediate and expert) based on the number of years of training that they had received. These levels were defined as follows. “Novice” dancers had received less than 5 years of continuous part-time training (*M* = 3 years; *SD* = 1.86). “Intermediate” dancers had received between 6 and 9 years of continuous part-time training (*M* = 8 years; *SD* = 1.43). Finally, “expert” dancers consisted of ballet or modern teachers who had gained at least 10 years of continuous part-time training and who had also obtained at least one dance teaching qualification with the imperial society of teachers of dance (ISTD) (*M* = 14 years; *SD* = 3.01).

### Materials

A short 15 s video of a *tendu* exercise (a short movement of the leg), from the grade 6 modern syllabus (Imperial Society of Teachers of Dance (ISTD), 2017) was used as the piece of choreography to be learned, performed and imagined by participants. *Tendu*, meaning “stretched out” in French, is a foot exercise aimed at warming up and strengthening the feet. This segment was deemed appropriate for three reasons. Firstly, it is drawn from grade 6, which precedes vocational standard examinations (such as intermediate foundation and intermediate). This means that the standard of the segment is between novice and intermediate level and just below that of teaching (expert) level. Secondly, the segment was chosen because both ballet and modern dance contain *tendus*. Therefore, as the sample consisted of mixed experience with the two styles, the exercise was deemed to be equally accessible to all participants. Finally, the segment was selected because, at 15 s of choreography, it provided a significant amount of data to be recorded by the Tobii glasses. It was also of a manageable length so that dancers could learn it under experimental conditions (i.e., it was not feasible to have a full hour long class per person where they would learn a longer piece). No participants had previously learned this particular *tendu* exercise so it was not within their behavioral repertoire. This short exercise required the dancers to carry out a *tendu* to the front, side and back on the right leg followed by the left, with a bend and stretch of both knees in between legs.

Two MI questionnaires were administered to participants before the learning, performing, and imagined movement conditions in order to assess their imagery abilities. These were the objective test of ability in movement imagery (TAMI; Madan and Singhal, 2013) and the subjective movement imagery questionnaire 3 (MIQ-3, Williams et al., 2012). The Tobii eye-tracking glasses (Tobii Technology, Stockholm, Sweden) were used to record participants’ pupil dilation (an index of attentional/mental effort) throughout the creative processes of learning, performing and imagining the piece of choreography. Finally, a stopwatch was used to record how long it took each participant to perform and then imagine the choreography, so that the differences between these times could be analyzed. All data collection took place in the same dance studio and the level of artificial light in the studio was kept constant, in order to avoid unwanted pupil dilation effects. The studio had mirrors on at least one wall of the room.

### Procedure

This research was first approved by the graduate research ethics committee, University College Dublin. The lead researcher contacted local dance schools where participants were recruited. Participation was voluntary and began only after the participant had provided informed consent. After such consent was obtained, the participants were provided with instructions and test materials for the TAMI and MIQ-3. Then, they completed these tests. In order to ensure anonymity, the participants were given an ID number which they wrote on their answer booklets and which was also used to label their pupillometry recordings. Upon completion of the questionnaires, participants wore the Tobii glasses and their pupils were calibrated. In order to do this, participants had to look at a light which was held at 9 different points at their eye level, about 2 m in front of them. The nine points are in the shape of a square, 3 points per line. The Tobii monitor is synced up to this light and indicates when calibration is complete at each point, what direction to move the light for the next point and when to move on to the next. It also indicates when calibration is complete.

Participants then watched a video of the *tendu* choreography three times, in order to learn it. They were told that they could mark the movements as they watched them if they wished. Such marking typically involves carrying out the choreography on a smaller scale rather than in full, perhaps using hand gestures to represent each movement. This is a common technique used by dancers when learning choreography (Nordin and Cumming, 2005) and increases the fidelity with which the learning condition represented a real-life, creative scenario. After three viewings, participants were asked to perform the piece in front of the mirrors, while still wearing the Tobii glasses so as to measure their attentional effort while performing. Their performance was also timed. In accordance with typical mental chronometry studies, participants were then asked to imagine themselves carrying out this same piece of choreography from a “third person” imagery perspective (i.e., they were asked to imagine it as if they were watching a video of themselves so it would be comparable to the learning condition). This was also timed so that timing of the performance and imagined movement conditions could be compared. Participants said “start” just before they began imagining it and “stop” when they were finished. They were then asked to remove the Tobii glasses and were provided with a debrief information sheet and thanked for their participation.

### Data Analysis

The data that were collected consisted of scores on the TAMI, MIQ-3 and also of times taken to perform and imagine the choreography. Scores on the TAMI were calculated using Madan and Singhal’s (2014) weighted scoring method, whereby more difficult questions gave a higher score than did easier questions. Scores on the MIQ-3 were calculated according to Williams et al.’s (2012) guidelines, whereby each self-report rating out of 7 was added up to reach a total score. Descriptive statistics were calculated and a reliability analysis was also conducted. Thirdly, the time taken to perform and imagine the choreography was recorded using a stop watch. Finally, pupillometry data were recorded by the Tobii eye-tracking glasses. These data were recorded in terms of percentages, whereby 100% is considered typical pupil size. Anything below 100% is seen as the pupil shrinking and above 100% is the amount that the pupil has dilated (Tobii Technology, Stockholm, Sweden, 2017). The typical pupil size, or baseline data, is recorded for each individual when they first put on the glasses and pupils are calibrated as the individual is required to fixate 9 different points. Data from all measures were inputted into SPSS (2017) (SPSS Inc., Chicago, Il, United States) for analysis.

Hypothesis 1, which proposed that there would be a statistically significant difference between each level of expertise in terms of scores on the TAMI and MIQ-3, was tested using two one-way between-groups ANOVAs. The independent variable was level of expertise (k = 3) and the dependent variables were scores on the TAMI and MIQ-3, respectively. Hypothesis 2, which proposed that there would be a statistically significant difference between time taken to perform the choreography and time taken to imagine performing the choreography, based on level of expertise, was also tested using a one-way ANOVA. In this case, the times taken to perform and imagine the choreography were subtracted from each other in order to calculate the difference. This score was then used as the dependent variable while level of expertise was the independent variable (*k* = 3). Hypothesis 3 predicted that there would be a statistically significant interaction between pupil dilation at the start, middle and end of the (a) learning, (b) performance, and (c) imagined movement conditions, based on level of expertise. To test this, percentage pupil dilation change was sampled at 33 Hz and was averaged over 1 s at the three time points. The starting point for the learning and performance conditions was at 0–1 s while the middle was 7.5–8.5 s and the end was at 14–15 s. As each participant imagined the movement in their own time, the time points for this condition started at 0–1 s, while middle was exactly halfway between this and when participants told the lead researcher that they were finished (which was the end point). In order to test this hypothesis, a three-way repeated measures ANOVA was carried out whereby the three factors were level of expertise (i.e., beginner, intermediate, and expert), time point (start, middle, and end) and task (learning, performing, or imagining the movement). Sphericity and Levene’s tests were conducted and relevant assumptions for the analyses were checked and met.

## Results

In order to test hypothesis 1 (as stated above), a set of one-way, between groups ANOVAs were conducted on participants’ imagery test scores. Although the apparent mean score differences would suggest an increase in performance on the TAMI and MIQ-3 as level of dance expertise increased (see Table 1), hypothesis 1 was not supported for the TAMI; *F*(2, 17) = 0.63, *p* = 0.55, η_p_^2^ = 0.077 or the MIQ-3; *F*(2, 17) = 2, *p* = 0.17, η_p_^2^ = 0.211. In order to test Hypothesis 2 (as stated above) imagined times were subtracted from movement times in order to calculate the difference. Then, a one-way ANOVA was carried out using this score for each participant. This hypothesis was also rejected, *F*(2,17) = 0.12, *p* = 0.88, η_p_^2^ = 0.016.

**Table 1:** Mean scores for the TAMI and MIQ.

Level of dance expertise	TAMI score (M)	MIQ score (M)
Beginner	15.33	65.16
Intermediate	16.33	74.15
Expert	17.33	76.33

**FIGURE 1:**
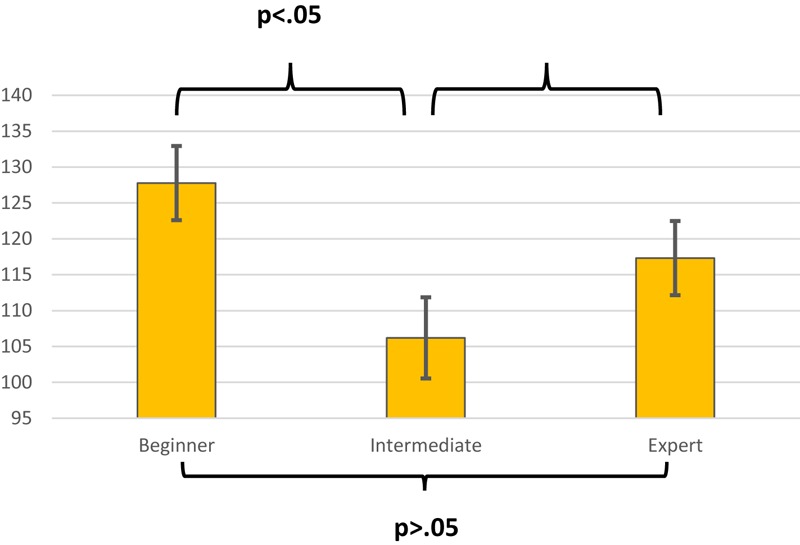
Estimated marginal mean pupillometry scores (with standard errors) and *p*-values for levels of expertise.

In order to test Hypothesis 3 (as stated above), a three-way repeated measures ANOVA was conducted. Hypothesis 3 was not supported as there was no statistically significant three-way interaction between dancers’ pupil dilation at the start, middle and end of each condition based on level of expertise, *F*(8,56) = 1.01.23, *p* = 0.193, η_p_^2^ = 0.173. Similarly, the two-way interactions and main effects of time or task were also not significant. However, a significant main effect of expertise was found in terms of their levels of pupil dilation, *F*(2,9) = 3.963, *p* = 0.043, η_p_^2^ = 0.362. *Post hoc* Scheffe multiple comparisons of pupillometry scores indicated that the beginners and experts did not significantly differ from each other in pupil dilation, but beginners had significantly higher pupil dilation than the intermediates (see Figure 1).

**FIGURE 2:**
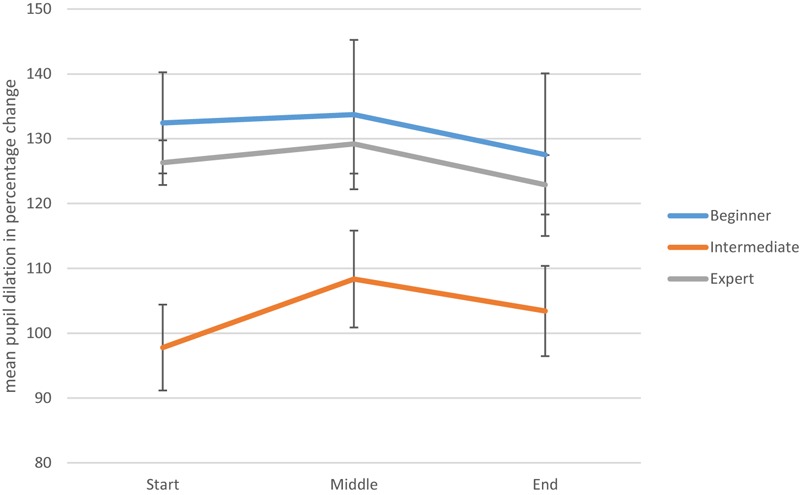
Mean pupil dilation levels throughout the performance condition (with standard error bars). This includes beginner, intermediate, and expert mean pupil dilation levels at three time points.

**FIGURE 3:**
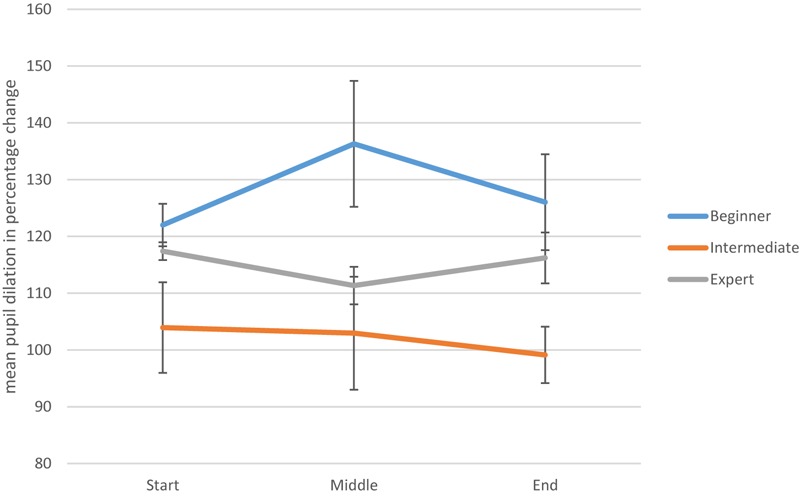
Mean pupil dilation levels throughout the imagined movement condition (with standard error bars). This includes beginner, intermediate, and expert mean pupil dilation levels at three time points.

A graphical portrayal of expertise-based differences in pupil dilation across three different time points was conducted for the performance condition (see Figure 2) and for the imagined movement condition (see Figure 3). Visual inspection of these two graphs suggest that the pattern of pupil dilation of experts over time is more consistent – particularly in the imagined condition - than for either the novice or intermediate performers. However, more fine-grained research is required to test the veracity of this observation. Additionally, an ANCOVA was carried out which explored whether or not the scores on the TAMI could account for the differences in pupillometry at each level of expertise. While TAMI score was not a significant covariate, *F*(1,13) = 2.47, *p* = 0.14, there was still difference between levels, *F*(2,13) = 3.709, *p* = 0.05. Thus, there is a significant difference between levels in terms of pupillometry scores, even when TAMI scores are controlled for.

## Discussion

The present study investigated the effect of expertise on dancers’ MI abilities and attentional effort while learning, performing, and imagining a piece of choreography. Whereas the TAMI and mental chronometry paradigm provided objective measures of the dancers’ MI abilities, the MIQ-3 provided a subjective index of these skills. Pupil dilation (as recorded by eye-tracking equipment) was used to measure the level of attentional effort exerted by the dancers during the learning, performance and imagined execution of choreographed movements.

Let us begin our interpretation of the results by considering the relationships among the different measures of MI. As this study is the first of its kind to assess MI in dancers using a combination of psychometric tests (the TAMI and MIQ-3) and mental chronometry measures, the results are somewhat exploratory in nature. Previously, Collet et al. (2011) found that the temporal congruence between a real and imagined movement was mediated by experience with the task in question. Unfortunately, our findings seem to contradict those of Collet et al. (2011). Some caution may be required when interpreting this inconsistency, however. This is so because the question of what precise aspect of MI the mental chronometry paradigm actually measures remains largely unresolved. Thus, Williams et al. (2015) raised the possibility that the MIQ-3 and chronometric tests may assess different components of MI. Specifically, they speculated that whereas the MIQ-3 may evaluate people’s ability to *generate* a motor image (i.e., creating the initial image in one’s minds eye), chronometric measures may assess people’s ability to *maintain* and *control* an image (i.e., retaining the image whilst also being able to manipulate different aspects of it). Collet et al. (2011) also make the point that it may be kinaesthetic imagery that relates to temporal congruence. As the participants in this study were specifically asked to create visual images, this may also explain the lack of significant differences.

Turning to the interpretation of dancers’ performance on the TAMI, it may be helpful to review the only previous published study in which an objective measure of MI was administered to dancers. In this study, Jola and Mast (2005) found that dancers scored higher than non-dancers on a measure of similar nature to the TAMI – the mental body transformation task (MBTT; Parsons, 1987). Extrapolating from this research, it may be possible that dancers are more proficient than non-dancers in manipulating body images but that their MI skills do not significantly improve with expertise. Although similarities can be drawn between the MBTT and the TAMI (as they both require participants to manipulate images of their bodies), the relationship between the two measures has not been analyzed to date. Accordingly, we do not know the extent to which these measures overlap in their assessment of MI skills. The fact that we found no evidence of expert-novice differences in dancers’ MIQ-3 scores is in line with results reported by Overby (1990). Recall that she found no significant differences between novice and experienced modern and ballet dancers on scores on the original MIQ. However, Williams et al. (2012) argued that the MIQ-3 is more likely than its predecessor to tap into differences in how easily one can generate movement images, due to the more specific demands it places on the participant (e.g., considering different image perspectives). One possible explanation for the absence of expert-novice differences in the present study is that the MIQ-3 may be too generic in its measurement of imagery ability. Thus, Pavlik and Nordin-Bates (2016) have argued that dance-specific imagery tools need to be developed because dancers use certain types of imagery (e.g., metaphorical imagery, where arms may be imagined as wings) that are not common among athletes. Clearly, it would be interesting to investigate the performance of dancers on dance-specific measures of MI. With regard to the pupillometry data (measuring attentional effort), a significant difference was discovered between experience levels at the start of the performance and imagined movement conditions. More specifically, this difference was detected between the beginners and intermediate-level dancers but not between the experts and intermediates or experts and beginners for both conditions. However, from inspection of Figure 2, the intermediates’ and experts’ pupils dilated slightly from the starting point to the middle stage, while the beginners’ pupils shrank slightly in comparison to the starting point. This difference may indicate that the beginners’ level of dilation at the start was due, in part, to the possibility that participants may have exerted some initial mental effort to work out the cognitive demands of this task. This could perhaps reflect the creative thinking processes required in order to navigate the cognitive challenge of co-ordinating dance movements, as described by Kaufman and Baer (2005) and Torrents et al. (2015). On the other hand, for the imagined movement condition, the beginners’ and intermediates’ pupils dilated between the start and 3.5 s, while the experts’ shrank slightly (see Figure 3). This may suggest that for beginners and intermediates it requires more mental effort to generate a motor image than it does for experts. Although there is currently no pupillometry data on dancers to compare this to, results in other areas of sport and performance psychology indicate that experts can generate motor images easier than can less skilled counterparts (Collet et al., 2011).

Let us now consider some methodological limitations of the present study. The first weakness concerns the absence of a kinematic performance measure for each dancer while attempting to master the choreographed movements. Although complex and time-consuming to implement, such a measure would have helped our study because it could have ensured that if any dancer had forgotten the choreography, the precise time point of this occurrence could have been noted and accounted for when analyzing subsequent pupillometry data. Additionally, it may have been useful to have a measure of how accurately each participant performed the choreography. This too could have been compared to pupillometry data for both the learning and performance conditions and could have contributed to our understanding of variances in pupil dilation across experience levels. It would be expected that the expert dancers who are more experienced in learning and performing, would perform and learn the choreography more accurately than would less skilled counterparts – which thus could subsequently affect levels of attentional effort. A second weakness concerns our interpretation of the pupil dilation data. According to Mathôt (2018), any information that activates the mind, or increases its “processing load” (Beatty, 1982; see also O’Shea and Moran, 2016), induces dilation of the pupil. In this paper, we have favored a mental effort-driven interpretation of pupil dilation. However, we must acknowledge that fluctuations in pupil size can occur for reasons other than as a function of the expenditure of mental effort. For example, Bouma and Baghuis (1971) speculated that they may be due simply to the waxing and waning of arousal. In a similar vein, Laeng et al. (2016) identified emotional engagement as a trigger for pupil dilation, whereas Hennessy and Amabile (2010) found emotion to be a hindering factor in creative processes. Unfortunately, as the present study lacked an independent measure of arousal and/or emotional engagement, we cannot exclude the possibility that these latter variables may have influenced our results. Nevertheless, our research is novel in being the first “process tracing” investigation of MI and attentional effort (as measured by pupil dilation) in dancers who are forced to engage in creative possibility thinking when learning and performing.

With regard to potentially fruitful directions for further psychological research on expert-novice differences in dance, several options are apparent. Firstly, future investigators of this topic may wish to include additional MI dimensions as imagery control (the ease with which a mental image can be manipulated by the person who creates it; Moran and Toner, 2017) and imagery accuracy or its “exactness of reference” (Denis, 1985). Secondly, it would be interesting to investigate the degree to which attentional effort affects the *accuracy* of dancers’ mental simulation and/or recall of dance movements, as the accuracy of performance may also reflect the extent to which the dancer could interpret and create these movements. Although MI and attentional effort may mediate the creative thinking required to learn and perform choreography, it may also be interesting to consider the effect of other factors in a dance setting which are known to interfere with creative thinking and the creation of one’s own choreography, for example, motivation and the environment (Hennessy and Amabile, 2010). Additionally, further research is required to explore the extent to which prolonged experience of learning and performing dance movements affects multi-sensory integration (the ability to combine information from different sensory modalities; Grunbaum and Schram Christensen, 2018).

To conclude, the present study suggests that there is a significant difference between beginner and intermediate dancers in levels of pupil dilation when faced with the task of performing and imagining a short piece of choreography. This finding is beneficial in understanding the cognitive demands which face the dancer, as well as the mechanisms which may underlie the creative thinking proposed necessary to the performing and imagining of choreography. The present study also paves way for further development of this research, such as administering several MI measures with dancers and comparing results, comparing pupil dilation with measures of arousal or performance appraisals and looking at what exact cognitive skills may vary with different levels of dance expertise.

## Ethics Statement

The study was carried out in accordance with the recommendations from University College Dublin’s Human Research Ethics Committee with written informed consent from all participants.

## Author Contributions

KC and AM contributed to the conception and design of the study. KC conducted the research and carried out the statistical analysis. KC and AM drafted sections of the first draft of the manuscript, while AM then edited, and critically revised it. BR contributed to the statistical analysis and interpretation of results in preparing the revised version of the manuscript and also to the responses to reviewers. All authors read and approved the final manuscript and agreed to be accountable for all aspects of the work.

## Conflict of Interest Statement

The authors declare that the research was conducted in the absence of any commercial or financial relationships that could be construed as a potential conflict of interest.

## References

[B1] Abraham A. Dunsky A. Dickstein R. (2017). The effect of motor imagery practice on elevé performance in adolescent female dance students: a randomized controlled trial. *J. Imag. Res. Sport. Phys. Act.* 12 10.1515/jirspa-2016-0006.

[B2] Beatty J. (1982). Task-evoked pupillary responses, processing load, and the structure of processing resources. *Psych. Bull.* 91 276–292. 10.1037/0033-2909.91.2.276 7071262

[B3] Beatty J. Lucero-Wagoner B. (2000). “The pupillary system,” in *Handbook of Psychophysiology* 2nd Edn. eds Cacioppo J. T. Tassinary L. G. Berntson G. G. (New York, NY: Cambridge University Press) 142–162.

[B4] Bouma H. Baghuis L. C. J. (1971). Hippus of the pupil: periods of slow oscillations of unknown origin. *Vis. Res.* 11 1345–1351. 10.1016/0042-6989(71)90016-2. 5148578

[B5] Burge W. K. Ross L. A. Amthor F. R. Mitchell W. G. Zotov A. Visscher K. M. (2013). Processing speed training increases the efficiency of attentional resource allocation in young adults. *Front. Hum. Neuro.* 7:684. 10.3389/fnhum.2013.00684. 24151461 PMC3799007

[B6] Chappell K. Craft A. Rolfe L. Jobbins V. (2009). Dance partners for creativity: choreographing space for co-participative research into creativity and partnership in dance education. *Res. Dance Educ.* 10 177–197. 10.1080/14647890903324147.

[B7] Collet C. Guillot A. Lebon F. MacIntyre T. Moran A. (2011). Measuring motor imagery using psychometric, behavioural, and psychophysiological tools. *Exerc. Sport Sci. Rev.* 39 85–92. 10.1097/JES.0b013e31820ac5e0 21206282

[B8] Cross E. S. Acquah D. Ramsey R. (2014). A review and critical analysis of how cognitive neuroscientific investigations using dance can contribute to sport psychology. *Int. Rev. Sport Exerc. Psychol.* 7 42–71. 10.1080/1750984X.2013.862564.

[B9] Cross E. S. Ticini L. F. (2012). Neuroaesthetics and beyond: new horizons in applying the science of the brain to the art of dance. *Phenomenol. Cogn. Sci.* 11 5–16. 10.1007/s11097-010-9190-y

[B10] Denis M. (1985). Visual imagery and the use of mental practice in the development of motor skills. *Can. J. App. Sport Sci.* 10 4s–16s. 3910301

[B11] Ericsson K. A. (2018). “Capturing expert thought with protocol analysis: concurrent verbalizations of thinking during experts’ performance on representative tasks,” in *The Cambridge Handbook of Expertise and Expert Performance* eds Ericsson K. A. Hoffman R. R. Kozbelt A. Williams A. M. (Cambridge: Cambridge University Press) 192–212. 10.1017/9781316480748.012

[B12] Fink A. Benedek M. Grabner R. H. Staudt B. Neubauer A. C. (2007). Creativity meets neuroscience: experimental tasks for the neuroscientific study of creative thinking. *Methods* 42 68–76. 10.1016/j.ymeth.2006.12.001. 17434417

[B13] Fink A. Grabner R. H. Benedek M. Neubauer A. C. (2006). Divergent thinking training is related to frontal electroencephalogram alpha synchronization. *Eur. J. Neurosci.* 23 2241–2246. 10.1111/j.1460-9568.2006.04751.x 16630071

[B14] Fink A. Graif B. Neubauer A. C. (2009). Brain correlates underlying creative thinking: EEG alpha activity in professional vs. novice dancers. *Neuroimage* 46 854–862. 10.1016/j.neuroimage.2009.02.036 19269335

[B15] Fink A. Woschnjak S. (2011). Creativity and personality in professional dancers. *Pers. Indiv. Diff.* 51 754–758. 10.1016/j.paid.2011.06.024

[B16] Fisher V. J. (2017). Unfurling the wings of flight: clarifying ‘the what’and ‘the why’of mental imagery use in dance. *Res. Dance Educ.* 18 252–272. 10.1080/14647893.2017.1369508.

[B17] Goldstein E. B. (2011). *Cognitive Psychology* 3rd Edn Belmont, CA: Wadsworth/Cengage.

[B18] Granholm E. Asarnow R. F. Sarkin A. J. Dykes K. L. (1996). Pupillary responses index cognitive resource limitations. *Psychophysiology* 33 457–461. 10.1111/j.1469-8986.1996.tb01071.x 8753946

[B19] Grunbaum T. Schram Christensen M. (2018), *Sensation of Movement* London: Routledge.

[B20] Guillot A. Collet C. (2005). Duration of mentally simulated movement: a review. *J. Mot. Behav.* 37 10–20. 10.3200/JMBR.37.1.10-20. 15642689

[B21] Guss-West C. Wulf G. (2016). Attentional focus in classical ballet: a survey of professional dancers. *J. Dance Med. Sci.* 20 23–29. 10.12678/1089-313X.20.1.23. 27025449

[B22] Hall C. R. Pongrac J. (1983). *Movement Imagery Questionnaire.* London: University of Western Ontario.

[B23] Hanakawa T. (2016). Organizing motor imageries. *Neuro. Res.* 104 56-63. 10.1016/j.neures.2015.11.003. 26602980

[B24] Heiland T. Rovetti R. (2013). Examining effects of franklin method metaphorical and anatomical mental images on college dancers’ jumping height. *Res. Dance Educ.* 14 141–161. 10.1080/14647893.2012.712105.

[B25] Hennessy B. A. Amabile T. M. (2010). Creativity. *Ann. Rev. Psychol.* 61 569–598. 10.1146/annurev.psych.093008.100416 19575609

[B26] Hess E. H. Polt M. (1964). Pupil size in relation to mental activity during simple problem solving. *Science* 140 1190–1192. 10.1126/science.143.3611.1190 17833905

[B27] Hétu S. Grégoire M. Saimpont A. Coll M. P. Eugène F. Michon P. E. (2013). The neural network of motor imagery: an ALE meta-analysis. *Neuro. Biobehav. Rev.* 37 930–949. 10.1016/j.neubiorev.2013.03.017. 23583615

[B28] Imperial Society of Teachers of Dance (ISTD) (2017). Grade 6 modern syllabus. London: ISTD.

[B29] James W. (1890). *The Principles of Psychology* Vol. 2 Cambridge, MA: Harvard University Press 10.1037/10538-000

[B30] Jeannerod M. (1994). The representing brain: neural correlates of motor intention and imagery. *Behav. Brain Sci.* 17 187–202. 10.1017/S0140525X00034026.

[B31] Jeannerod M. (2001). Neural simulation of action: a unifying mechanism for motor cognition. *Neuroimage.* 14 103–109. 10.1006/nimg.2001.0832. 11373140

[B32] Jeannerod M. (2006). *Motor Cognition.* New York, NY: Oxford University Press 10.1093/acprof:oso/9780198569657.001.0001

[B33] Jola C. Mast F. W. (2005). Mental object rotation and egocentric body transformation: two dissociable processes? *Spatial Cogn. Comput.* 5 217–237. 10.1080/13875868.2005.9683804.

[B34] Kahneman D. (1973). *Attention and Effort.* New York, NY: Prentice-Hall.

[B35] Kasof J. (1997). Creativity and breadth of attention. *Creat. Res. J.* 10 303–315. 10.1207/s15326934crj1004_2

[B36] Kaufman J. C. Baer J. (eds.). (2005). *Creativity Across Domains: Faces of the Muse.* London: Psychology Press 10.4324/9781410611925

[B37] Laakso A. (2011). Embodiment and development in cognitive science. *Cogn. Brain Behav. Interdisc. J.* 4 409–425.

[B38] Laeng B. Eidet L. M. Sulutvedt U. Panksepp J. (2016). Music chills: the eye pupil as a mirror to music’s soul. *Consc. Cogn.* 44 161–178. 10.1016/j.concog.2016.07.009. 27500655

[B39] Madan C. R. Singhal A. (2013). Introducing TAMI: an objective test of ability in movement imagery. *J. Mot. Behav.* 45 153–166. 10.1080/00222895.2013.763764. 23557260

[B40] Madan C. R. Singhal A. (2014). Improving the TAMI for use with athletes. *J. Sport. Sci.* 32 1351–1356. 10.1080/02640414.2014.889847. 24669880

[B41] Mathôt S. (2018). Pupillometry: psychology, physiology, and function. *J. Cogn.* 16 1–23, 10.5334/joc.18 PMC6634360 31517190

[B42] Mathôt S. Van der Stigchel S. (2015). New light on the mind’s eye: the pupillary light response as active vision. *Curr. Dir. Psychol. Sci.* 24 374–378. 10.1177/0963721415593725. 26494950 PMC4601080

[B43] Moran A. (1996). *The Psychology of Concentration in Sport Performers: A Cognitive Analysis.* Hove: Psychology Press.

[B44] Moran A. Guillot A. MacIntyre T. Collet C. (2012). Re-imagining motor imagery: building bridges between cognitive neuroscience and sport psychology. *Br. J. Psychol.* 103 224–247. 10.1111/j.2044-8295.2011.02068.x 22506748

[B45] Moran A. Quinn A. Campbell M. Rooney B. Brady N. Burke C. (2016). Using pupillometry to evaluate attentional effort in quiet eye: a preliminary investigation. *Sport Exerc. Perform. Psychol.* 5:365 10.1037/spy0000066Issn.

[B46] Moran A. Toner J. (2017). *A Critical Introduction to Sport Psychology* 3rd Edn London: Routledge 10.4324/9781315657974

[B47] Murphy P. R. O’Connell R. G. O’Sullivan M. Robertson I. H. Balsters J. H. (2014). Pupil diameter covaries with BOLD activity in human locus coeruleus. *Hum. Brain Map.* 35 4140–4154. 10.1002/hbm.22466. 24510607 PMC6869043

[B48] Nordin S. M. Cumming J. (2005). Professional dancers describe their imagery: where, when, what, why, and how. *Sport Psychol.* 19 395–416. 10.1123/tsp.19.4.395.

[B49] Nordin S. M. Cumming J. (2007). Where, when, and how: a quantitative account of dance imagery. *Res. Quart. Exerc. Sport* 78 390–395. 10.1080/02701367.2007.10599437. 17941544

[B50] O’Shea H. Moran A. (2016). Chronometric and pupil-size measurements illuminate the relationship between motor execution and motor imagery in expert pianists. *Psychol. Music* 44 1289–1303. 10.1177/0305735615616286.

[B51] O’Shea H. Moran A. (2017). Does motor simulation theory explain the cognitive mechanisms underlying motor imagery? a critical review. *Front. Hum. Neuro.* 11:72. 10.3389/fnhum.2017.00072 28261079 PMC5313484

[B52] Overby L. Y. (1990). A comparison of novice and experienced dancers’ imagery ability. *J. Men. Imag.* 14 173–184.

[B53] Overby L. Y. Dunn J. (2011). The history and research of dance imagery: implications for teachers. *IADMS Bull. Teach.* 3 9–11.

[B54] Parsons L. M. (1987). Imagined spatial transformations of one’s hands and feet. *Cogn. Psychol.* 19 178–241. 10.1016/0010-0285(87)90011-9. 3581757

[B55] Pavlik K. Nordin-Bates S. (2016). Imagery in dance: a literature review. *J. Dance Med. Sci.* 20 51–63. 10.12678/1089-313X.20.2.51. 27245944

[B56] Piquado T. Isaacowitz D. Wingfield A. (2010). Pupillometry as a measure of cognitive effort in younger and older adults. *Psychophysiology* 47 560–569. 10.1111/j.1469-8986.2009.00947.x 20070575 PMC2867103

[B57] Porter G. Troscianko T. Gilchrist I. D. (2007). Effort during visual search and counting: insights from pupillometry. *Quart. J. Exp. Psychol.* 60 211–229. 10.1080/17470210600673818 17455055

[B58] Sarter M. Gehring W. J. Kozak R. (2006). More attention must be paid: the neurobiology of attentional effort. *Brain Res. Rev.* 51 145–160. 10.1016/j.brainresrev.2005.11.002 16530842

[B59] Shepard S. Metzler D. (1988). Mental rotation: effects of dimensionality of objects and type of task. *J. Exper. Psychol. Hum. Percept. Perform.* 14 3–11. 10.1037/0096-1523.14.1.3 2964504

[B60] Simonton D. K. Damian R. I. (2013). “Creativity,” in *Handbook of Cognitive Psychology* ed Reisberg D. (New York, NY: Oxford University Press) 795–807.

[B61] Smith E. E. Kosslyn S. M. (2007). *Cognitive Psychology: Mind and Brain (International Edition).* Upper Saddle River, NJ: Pearson.

[B62] SPSS (2017). *IBM SPSS Statistics for Windows, Version 25.0*. Armonk, NY: IBM Corp.

[B63] Stevens C. Winskel H. Howell C. Vidal L. M. Latimer C. Milne-Home J. (2010). Perceiving dance schematic expectations guide experts’ scanning of a contemporary. *J. Dance Med. Sci.* 14 19–25 10.1136/bmj.c889 20214851

[B64] Torrents C. Ric Á. Hristovski R. (2015). Creativity and emergence of specific dance movements using instructional constraints. *Psychol. Aesth. Creat. Arts* 9:65 10.1037/a0038706

[B65] Unsworth N. Robison M. K. (2015). Individual differences in the allocation of attention to items in working memory: evidence from pupillometry. *Psychon. Bull. Rev.* 22 757–765. 10.3758/s13423-014-0747-6 25324180

[B66] Weisberg R. W. (2018). “Expertise and structured imagination in creative thinking: reconsideration of an old question,” in *The Cambridge Handbook of Expertise and Expert Performance* eds Ericsson K. A. Hoffman R. W. Kozbelt A. Williams A. M. (Cambridge: Cambridge University Press) 812–834. 10.1017/9781316480748.041

[B67] Williams A. M. Ericsson K. A. (2005). Perceptual-cognitive expertise in sport: some considerations when applying the expert performance approach. *Hum. Mov. Sci.* 24 283–307. 10.1016/j.humov.2005.06.002 16095739

[B68] Williams S. E. Cumming J. Ntoumanis N. Nordin-Bates S. M. Ramsey R. Hall C. (2012). Further validation and development of the movement imagery questionnaire. *J. Sport Exer. Psychol.* 34 621–646. 10.1123/jsep.34.5.621 23027231

[B69] Williams S. E. Guillot A. Di Rienzo F. Cumming J. (2015). Comparing self-report and mental chronometry measures of motor imagery ability. *Eur. J. Sport Sci.* 15 703–711. 10.1080/17461391.2015.1051133 26313631

